# Frequency-Reconfigurable Wide-Angle Terahertz Absorbers Using Single- and Double-Layer Decussate Graphene Ribbon Arrays

**DOI:** 10.3390/nano8100834

**Published:** 2018-10-14

**Authors:** Longfang Ye, Fang Zeng, Yong Zhang, Xiong Xu, Xiaofan Yang, Qing Huo Liu

**Affiliations:** 1 Institute of Electromagnetics and Acoustics, Department of Electronic Science, Xiamen University, Xiamen 361005, China; fangzeng@stu.xmu.edu.cn; 2 EHF Key Laboratory of Fundamental Science, University of Electronic Science and Technology of China, Chengdu 611731, China; yongzhang@uestc.edu.cn; 3 State Key Laboratory of Complex Electromagnetic Environment Effects on Electronics and Information System, Luoyang 471003, China; xuxiong2008@foxmail.com (X.X.); xiaofan_uestc@sina.com (X.Y.); 4 Department of Electrical and Computer Engineering, Duke University, Durham, NC 27708, USA; qhliu@duke.edu

**Keywords:** terahertz, graphene, frequency reconfigurable absorber, surface plasmons

## Abstract

We propose and numerically demonstrate two novel terahertz absorbers made up of periodic single- and double-layer decussate graphene ribbon arrays. The simulated results show that the proposed absorbers have narrowband near-unity terahertz absorption with ultra-wide frequency reconfiguration and angular stability. By tuning the Fermi level of graphene ribbons, the over 90% absorbance peak frequency of the absorber with single-layer graphene structure can be flexibly adjusted from 6.85 to 9.85 THz for both the transverse magnetic (TM) and transverse electric (TE) polarizations. This absorber with single-layer graphene demonstrates excellent angular stability with the absorbance peaks of the reconfigurable absorption bands remaining over 99.8% in a wide angle of incidence ranging from 0 to 70°. The tuning frequency can be significantly enhanced by using the absorber with double-layer graphene structure from 5.50 to 11.28 THz and 5.62 to 10.65 THz, approaching two octaves under TM and TE polarizations, respectively. The absorbance peaks of the reconfigurable absorption band of this absorber for both polarizations maintain over 70%, even at a large angle of incidence up to 70°. Furthermore, an analytical fitting model is also proposed to accurately predict the absorbance peak frequencies for this variety of absorbers. Benefitting from these attractive properties, the proposed absorber may have great potential applications in tunable terahertz trapping, detecting, sensing, and various terahertz optoelectronic devices.

## 1. Introduction

Terahertz waves generally refer to electromagnetic waves with a frequency of 0.1–10 THz. Terahertz technology has broad applications in terahertz communication, spectroscopy, detecting, sensing, and imaging [1,2,3,4]. THz metamaterial absorbers (MAs) have aroused increasing attention since Landy et al. proposed a perfect MA in 2008 [5]. For many applications, frequency-tunable narrowband MAs would be preferred over typical broadband MAs, owing to their ability to absorb given frequency resonance without influencing neighboring spectrums. For conventional MAs, the absorption characteristics can only be manipulated by changing the resonator geometry or the surrounding medium [6]. Recently, by integrating varactors [7], semiconductors [8], microelectromechanical systems (MEMS) [9], diodes [10], and liquid crystals [11], various tunable MAs with frequency, bandwidth, and strength adjustability have been achieved. However, such tunable absorbers usually have very limited frequency tuning range and also require large external controls and complicated manufacturing techniques. 

Graphene is a monatomic carbon layer two-dimensional material that has attracted a great deal of attention in the past decade because of its unique electrical performance, low intrinsic loss, and photonic properties [12]. Since graphene possesses the ability to support long propagation and strongly localized surface plasmon polaritons (LSPPs) in the terahertz and infrared regions, it has become an excellent electromagnetic film material widely used in various terahertz components and devices [13,14,15]. To date, many graphene-based MAs have been proposed and investigated in a wide frequency spectrum ranging from microwave to ultraviolet light [16,17,18,19,20]. Via the chemical or electrostatic doping of graphene, both strong absorption and flexible tunability can be easily achieved. Especially, as a new type of MAs with broad potential applications in electromagnetic wave sensing, modulation, detection, and stealth, graphene-based frequency reconfigurable absorbers have recently attracted increasing attention. For example, Ning et al. proposed a tunable mid-infrared absorber made up of a graphene–metal nanostructure with angular-insensitive absorption within −12° to 12° [21]. Meanwhile, by tuning the graphene Fermi level from 0.2 to 0.8 eV, the absorbance peak wavelength (frequency) shifts from 9969 nm (30.1 THz) to 9318 nm (32.2 THz), corresponding to the relative frequency tuning range (RFTR) of 7%, where the RFTR is defined as the absolute absorbance peak frequency tuning range over the lowest absorbance peak frequency, (*f*_H_ − *f*_L_)/*f*_L_ × 100%. Li et al. demonstrated a tunable mid-infrared narrowband absorber using parallel double-layer graphene ribbons with an RFTR of 18.7% from 22.73 to 27 THz and a wide angular stability of 60° [22]. Wu investigated a tunable absorber using a dielectric grating-graphene-Bragg grating structure with an RFTR of 1.1% from 8.92 to 9.02 THz [23]. He et al. presented an active graphene-based terahertz MA with an RFTR of 20.6% from 1.31 to 1.58 THz [24]. Zhang et al. proposed a graphene-based tunable MA with an RFTR of 14.9% from 0.94 to 1.08 THz [25]. An enhanced angular stability up to 70° (50°) was achieved in that absorber for transverse magnetic (TM) (transverse electric, TE) polarized terahertz waves at 0.94 THz, but the insensitive angle of incidence was drastically decreased to 25° as the absorption band shifted to the higher-frequency end at 1.08 THz. In addition, dual-band tunable terahertz and mid-infrared MAs with RFTRs of 31.4% and 19.5% were also achieved [26,27]. Despite the recent progress in tunable MAs, most of these graphene-based absorbers still suffer from the disadvantages of small angular stability and very limited RFTR range, usually less than 35%. How to achieve tunable graphene-based absorbers with larger RFTR and wider angular stability remains a challenge.

To address this issue, we propose a new variety of frequency-reconfigurable narrowband near-unity terahertz absorbers with significantly enhanced RFTR and angular stability using single/double-layer decussate graphene ribbon arrays (S/DLDGRAs). To demonstrate the properties and mechanisms of the absorbers, we present the geometric configurations and study their performance, including terahertz absorbance spectra, effective impedance characteristics, field distributions, frequency reconfiguration, and angular stability of the proposed absorbers. It was found that by tuning graphene Fermi level, the RFTR for the over 90% absorbance peak frequency of the proposed absorber with SLDGRA reached 43.8%, ranging from 6.85 to 9.85 THz for both TM and TE polarizations under normal incidence. The RFRT for the over 90% absorbance peak frequency of the absorber with DLDGRA could be further extended to 105.1% (89.5%), ranging from 5.50 (5.62) to 11.28 (10.65) THz, approaching two octaves for the TM (TE) polarization under normal incidence. The absorbance peaks of the different absorption bands for both absorbers maintained over 70% in a wide range of angle of incidence from 0° to 70°. Furthermore, we propose a simple analytical fitting model to accurately predict the reconfigurable absorbance frequencies of the absorbers. Finally, we systematically draw the conclusion of the whole work.

## 2. Geometric Configurations of the Absorbers

**Figure 1:**
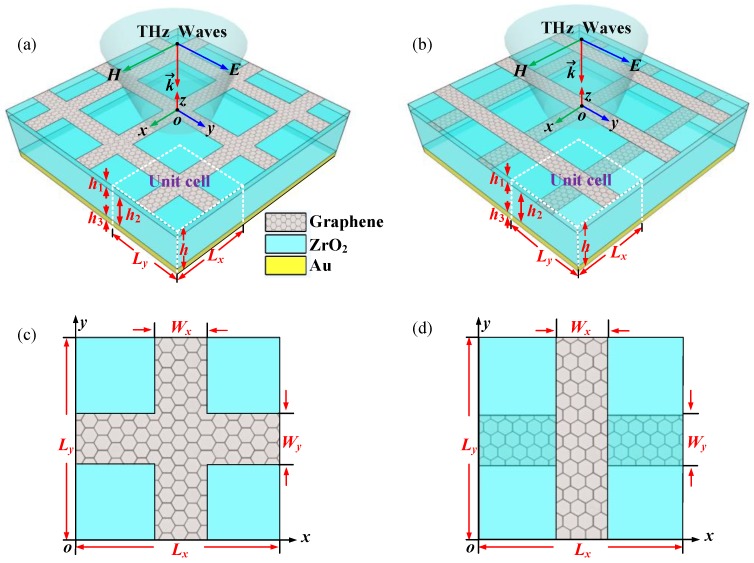
Schematic illustrations of the proposed frequency-reconfigurable wide-angle terahertz absorbers. (**a**) The absorber with a single-layer decussate graphene ribbon array (SLDGRA); (**b**) The absorber with double-layer decussate graphene ribbon arrays (DLDGRAs); (**c**) top view of the unit cell of the proposed absorber with SLDGRA; (**d**) top view of the unit cell of the proposed absorber with DLDGRA. The geometric parameters of the absorbers were set as *L_x_* = *L_y_* = 4 µm, *W_x_* = *W_y_* = 0.9 μm, *h*_1_ = 1 μm, *h*_2_ = 4 μm, *h*_3_ = 1 μm.

The schematic of the proposed frequency-reconfigurable wide-angle terahertz absorbers with S/DLDGRAs is illustrated in Figure 1. The absorber with a SLDGRA is made up of a periodic graphene ribbon array, a ZrO_2_ dielectric layer, and a gold (Au) reflection layer from the top to the bottom. The absorber with DLDGRAs can be interpreted as the transformation of the absorber with a SLDGRA by separating two graphene ribbon arrays into different layers with a distance of *h*_1_ while keeping the identical thickness of the dielectric layer. Here, we display the top views of the unit cells of the proposed absorbers with S/DLGRAs in Figure 1c,d, respectively. In the terahertz region, the surface conductivity of the graphene is calculated by the Kubo formula as *σ*_g_ = *σ*_intra_ + *σ*_inter_ (Unit: S) [28,29], where the intraband contribution *σ*_intra_ and interband contribution *σ_inter_* are given by:

(1) $$\sigma_{intra}(\omega ,\mu_{c},\Gamma ,T_{0})=\frac{je^{2}}{\pi \hbar^{2}\left(\omega -j2\Gamma \right)}\overset{\infty }{\underset{0}{\int }}\left(\frac{\partial f_{d}\left(\xi ,\mu_{c},T_{0}\right)}{\partial \xi }-\frac{\partial f_{d}\left(-\xi ,\mu_{c},T_{0}\right)}{\partial \xi }\right)\xi d\xi ,$$

(2) $$\sigma_{inter}(\omega ,\mu_{c},\Gamma ,T_{0})=-\frac{je^{2}\left(\omega -j2\Gamma \right)}{\pi \hbar^{2}}\overset{\infty }{\underset{0}{\int }}\frac{f_{d}\left(-\xi ,\mu_{c},T_{0}\right)-f_{d}\left(\xi ,\mu_{c},T_{0}\right)}{{\left(\omega -j2\Gamma \right)}^{2}-4\xi /\hbar^{2}}d\xi ,$$

where $f_{d}(\xi ,\mu_{c},T)={\left(e^{\left(\xi -\mu_{c}\right)/k_{B}T}+1\right)}^{-1}$ is the Fermi–Dirac distribution, *ω* is the radian frequency, *μ*_c_ is the chemical potential or Fermi level, *T*_0_ is the temperature, *Γ* is the phenomenological scattering rate, *Γ* = 2*τ*^−1^, *τ* is the relaxation time, *e* is the charge of an electron, *ξ* is energy, *ћ* is the reduced Plank’s constant, and *k*_B_ is Boltzmann’s constant. The relative dielectric constant of ZrO_2_ was set as *ε*_r_ = 4.41 [30,31] and the relative dielectric constant of Au was obtained by the Drude Model [32]. In the absorber designs, *L*_x_ and *L*_y_ are the periods of the unit cell, and *W*_x_ and *W*_y_ are the widths of the graphene ribbons in *x* and *y* directions, respectively. The thickness of the upper and lower ZrO_2_ spacers, the Au reflecting layer, and the total thickness of the absorbers were set as *h*_1_, *h*_2_, *h*_3_, and *h* = *h*_1_ + *h*_2_ + *h*_3_, respectively. In this study, we assumed the parameters as *L*_x_ = *L*_y_ = 4 µm, *W*_x_ = *W*_y_ = 0.9 μm, *h*_1_ = 1 μm, *h*_2_ = 4 μm, *h*_3_ = 1 μm, *T*_0_ = 300 K, *τ* = 0.6 ps [33], respectively. In the numerical simulations, graphene ribbons were modeled as equivalent two-dimensional surface impedance layers with *Z_g_ =* 1*/σ_g_* without thickness [34,35] and the finite element method-based frequency domain solver of the commercial software CST Studio was used to study the properties of the absorbers. The absorbance of the absorbers is defined as *A* = 1−*T*–*R*, where reflectance *R* = |*S*_11_|^2^, transmission *T* = |*S*_21_|^2^, and *S* denotes the scattering parameter, respectively [20]. In addition, the theoretical and experimental available Fermi level *μ*_c_ of graphene can be easily tuned from 0 to 1.0 eV via chemical or electrostatic doping [29,36,37], making it possible to achieve flexible control of the absorbance properties of the absorbers.

## 3. Results and Discussion

In this section, we first study terahertz absorbance spectra, frequency reconfiguration and angular stability of the proposed absorbers with SLDGRA. Then, to demonstrate a wider frequency reconfiguration, we present and investigate the properties of the absorbers with DLDGRA. Furthermore, we also proposed a simple analytical fitting model to accurately predict the reconfigurable absorbance peak frequencies of this variety of absorbers.

### 3.1. The Terahertz Absorption Performance of the Absorber with a SLDGRA

**Figure 2:**
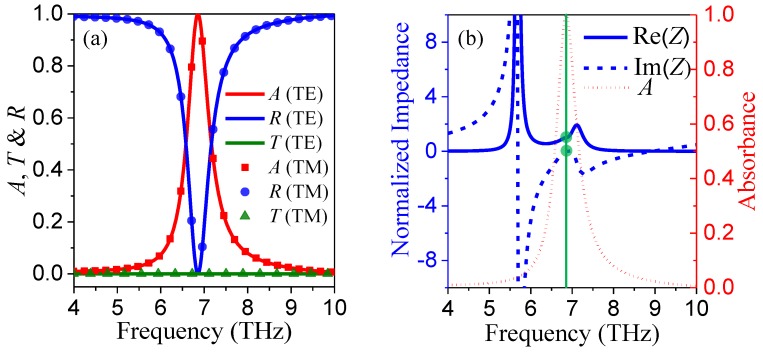
(**a**) Simulated absorbance (*A*), transmission (*T*), and reflectance (*R*) of the proposed absorbers with SLDGRAs for both transverse magnetic (TM) and transverse electric (TE) polarizations at *μ*_c_ = 0.3 eV under normal terahertz incidence; (**b**) The real and imaginary parts of the retrieved effective impedance (*Z*) of the absorber for both TM and TE polarization, where the black and red curves indicate the Re(*Z*) and Im(*Z*), respectively, and the vertical green solid cut-line denotes the frequency of absorbance peak at 6.85 THz.

**Figure 3:**
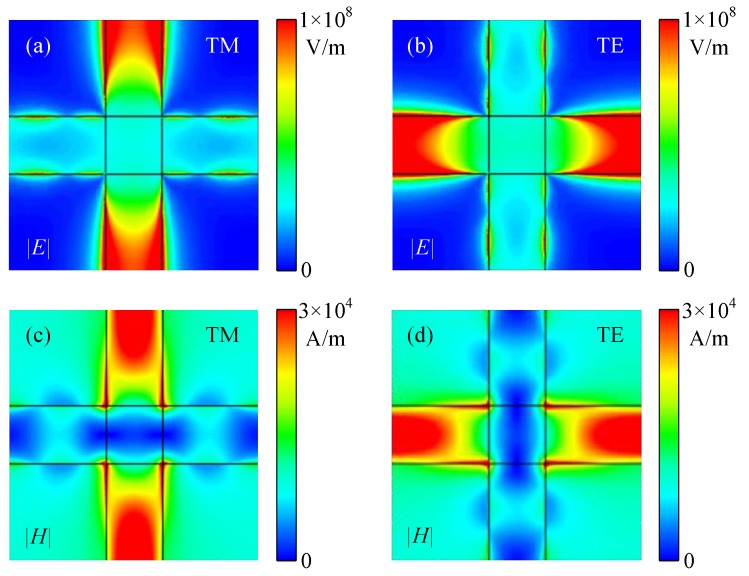
(**a**–**d**) are the electric field distributions, |*E*|, and magnetic field distributions, |*H*|, for TM and TE polarizations of the absorbers with SLDGRAs along the cut-plane on the top graphene layer at 6.85 THz with *μ*_c_ = 0.3 eV under normal terahertz incidence, respectively.

First, we investigated the terahertz absorption performance of the proposed absorber with a SLDGRA under normal incidence. The identical geometric parameters are shown in Figure 1a. This structure can be regarded as an asymmetric Fabry–Perot-like resonant cavity. Figure 2a shows the simulated absorbance *A*, transmission *T*, and reflectance *R* for both TE and TM polarizations with *μ*_c_ = 0.3 eV under normal terahertz incidence (*θ* = 0°). It is clear that excellent narrow-band terahertz absorption was achieved with the absorbance peak approaching 100% at 6.85 THz. Because of the axisymmetric unit cell, as shown in Figure 1c, this absorber demonstrated polarization independence for the TE- and TM-polarized incident terahertz waves under normal incidence. The underlying absorption mechanism can be interpreted by the effective medium theory [38], where the normalized effective impedance is given by $Z=\sqrt{\left({\left(1+S_{11}\right)}^{2}-S_{21}{}^{2}\right)/\left({\left(1-S_{11}\right)}^{2}-S_{21}{}^{2}\right)}$. As shown in Figure 2b, the blue solid and dashed curves denote the real and imaginary parts of *Z*, respectively. We observed that for upper green point, Re(*Z*) = 1.0004, and for the lower green point, Im(*Z*) = −0.00114, at the absorbance peak frequency of 6.85 THz, implying the impedance of the absorber matched the free space well. Therefore, since the extremely low reflectance resulting from smooth impedance matching together with zero transmission enabled by the thick gold layer, the near-unity absorbance of the absorber was achieved. Figure 3a–d display the electric field distributions, |*E*|, and magnetic field distributions, |*H*|, for the TM and TE polarizations along the cut-plane on the top graphene layer at 6.85 THz. The localized surface plasmon polaritons (LSPPs) were excited and strongly confined on the graphene ribbons, leading to ultra-high terahertz absorption. As expected from the symmetric configuration and polarization-independent property, the |*E*| and |*H*| for TM polarization were exactly 90° shifted from that of the TE polarization. Therefore, this absorber with a SLDGRA showed a clear independence of polarization, which has a significant advantage over the polarization-sensitive absorber with single graphene ribbon array. 

**Figure 4:**
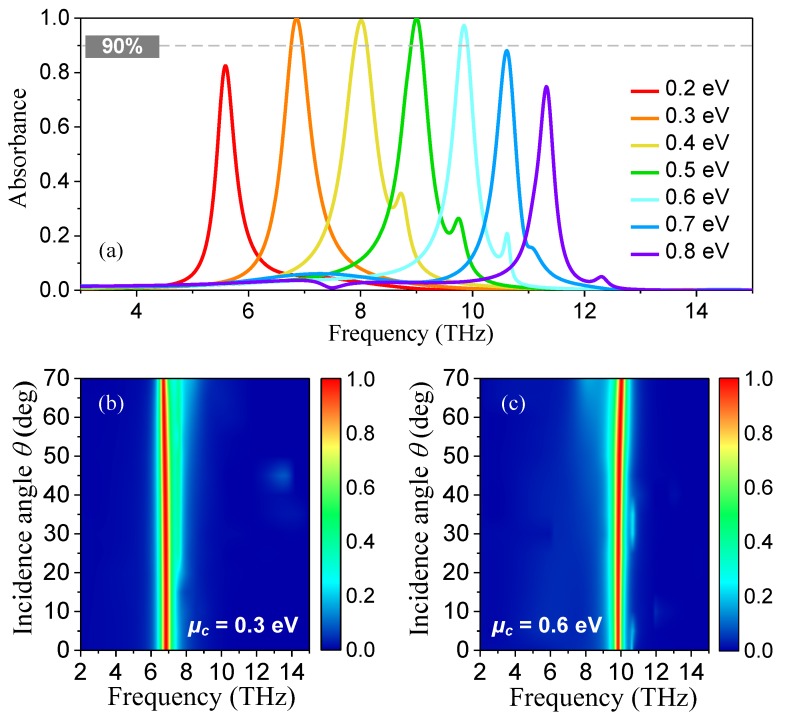
(**a**) Simulated absorption spectra for both TM and TE polarizations under normal incidence with *μ*_c_ ranging from 0.2 to 0.8 eV; (**b**,**c**) are the absorbance spectra as a function of incidence angle and operating frequency with *μ*_c_ fixed as 0.3 and 0.6 eV, respectively.

To demonstrate the properties of wide frequency reconfiguration and angular stability, we studied the dependence of Fermi level *μ*_c_ and angle of incidence *θ* on the absorbance spectra. Figure 4a depicts the absorbance spectra for both TM and TE polarizations under normal incidence with various *μ*_c_. It was found that changing the *μ*_c_ of the graphene ribbon had a significant influence on the absorption frequency band. As *μ*_c_ increased from 0.2 to 0.8 eV, the absorbance peak frequency band red-shifted accordingly from 5.58 to 11.32 THz. Especially, good frequency reconfiguration characteristics were clearly observed. As *μ*_c_ increased from 0.3 to 0.6 eV, the over 90% absorbance peak frequency varied from 6.85 to 9.85 THz, demonstrating a wide absolute frequency tuning range of 3 THz with the RFTR of 43.8%, which was larger than the recently reported works [21,22,23,24,25,26,27]. Figure 4b,c display the simulated absorbance spectra as a function of incidence angle and operating frequency with *μ*_c_ of 0.3 and 0.6 eV, for the first and the last absorption bands with over 90% peak absorbance, respectively. The results showed that the absorber also possessed an excellent angular stability for narrowband terahertz absorption characteristics. Owing to the wide-angle localized resonance of SPPs in the SLDGRA structure, the peak absorbance remained over 99.8%, even at a large oblique incidence angle of 70° for both cases. This absorber with wide frequency reconfiguration, absolute polarization independence, and excellent angular stability may have significant potential applications in the terahertz regime.

### 3.2. Terahertz Absorption Performance of the Absorber with DLDGRAs

**Figure 5:**
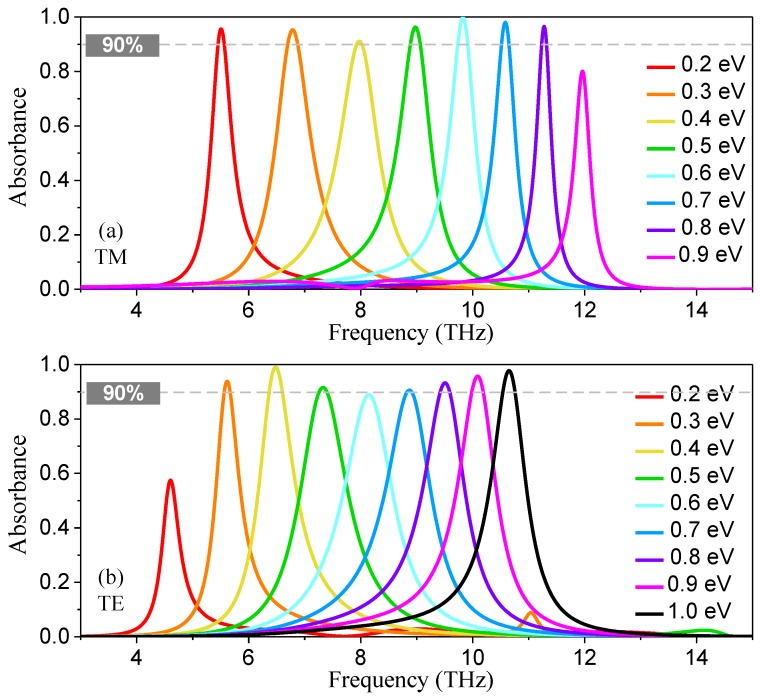
Simulated absorbance spectra of the proposed absorber with DLDGRAs with different *μ*_c_ under normal terahertz incidence. (**a**) Absorbance spectra for TM polarization with *μ*_c_ ranging from 0.2 to 0.9 eV; (**b**) absorbance spectra for TE polarization with *μ*_c_ ranging from 0.2 to 1.0 eV.

To further enhance the absorbance peak frequency tuning range, we propose the absorber with DLDGRAs, as shown in Figure 1b,d. The ZrO_2_ separation (*h*_1_) of the double-layer decussate graphene ribbon arrays was set as 1 µm while the rest of the geometric parameters of this absorber were fixed to be the same as the absorber with a SLDGRA. The simulated absorbance spectra for TM and TE polarizations with different *μ*_c_ at normal terahertz incidence are displayed in Figure 5a,b, respectively. Clearly, ultra-wide frequency tuning of the narrowband terahertz absorption was obtained by varying *μ*_c_. For TM polarizations, the reconfigurable over 90% absorbance peak frequency significantly switched from 5.50 to 11.28 THz, corresponding an enhanced RFTR of 105.1%, as the *μ*_c_ increased from 0.2 to 0.8 eV. For TE polarization, the reconfigurable over 90% absorbance peak frequency switched from 5.62 to 10.65 THz, corresponding to a RFTR of 89.5%, as the *μ*_c_ varied from 0.3 to 1.0 eV. Compared with the RFTR of the absorber with a SLDGRA, ultra-wide frequency reconfiguration with 2× more RFTR was achieved in the absorber with DLDGRAs. 

**Figure 6:**
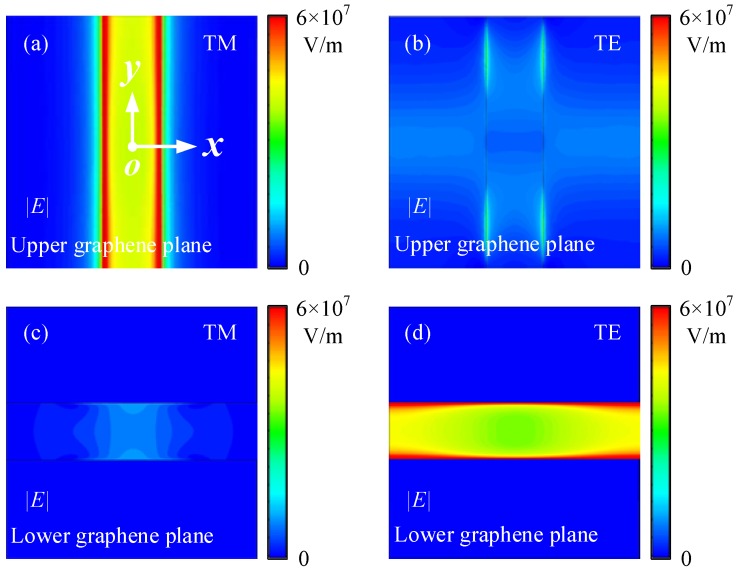
Electric field distributions of the absorber with DLDGRAs at *μ*_c_ = 0.3 eV under normal incidence, where (**a**,**c**) are respectively the |*E*| for the TM polarization along the upper and lower graphene planes at the absorbance peak frequency of 6.81 THz; (**b**,**d**) are the |*E*| for the TE polarization along the upper and lower graphene planes at the absorbance peak frequency of 5.62 THz, respectively.

Next, it is remarkable that this absorber was no longer polarization-independent because of the non-axisymmetric DLDGRA structure. To better understand this characteristic, we depict the simulated electric field distributions for TM and TE polarizations along the cut-planes on upper and lower graphene layers with *μ*_c_ = 0.3 eV under normal incidence. For TM polarization, |*E*| distributions of the LSPPs at the absorbance peak frequency of 6.81 THz along upper and lower graphene plane are shown in Figure 6a,c, respectively. It was clearly observed that |*E*| were mainly confined around upper-layer graphene ribbon edges. Therefore, the terahertz absorption for the TM polarization was mainly caused by LSPPs located at the upper-layer graphene ribbons. Similarly, the electric distributions for the TE polarization at the absorbance peak frequency of 5.62 THz along upper and lower graphene plane are shown in Figure 6b,d. The LSPPs were strongly excited and concentrated around the lower graphene ribbons, leading to strong terahertz absorption. Clearly, the absorption is largely dependent on the localized resonance of SPPs in the individual layer of graphene ribbons rather than the interplay of SPPs between upper- and lower-layer graphene ribbons. The different field distributions of LSPPs determine the different terahertz absorbance spectra of the proposed absorber with DLDGRAs under TM and TE polarizations. 

**Figure 7:**
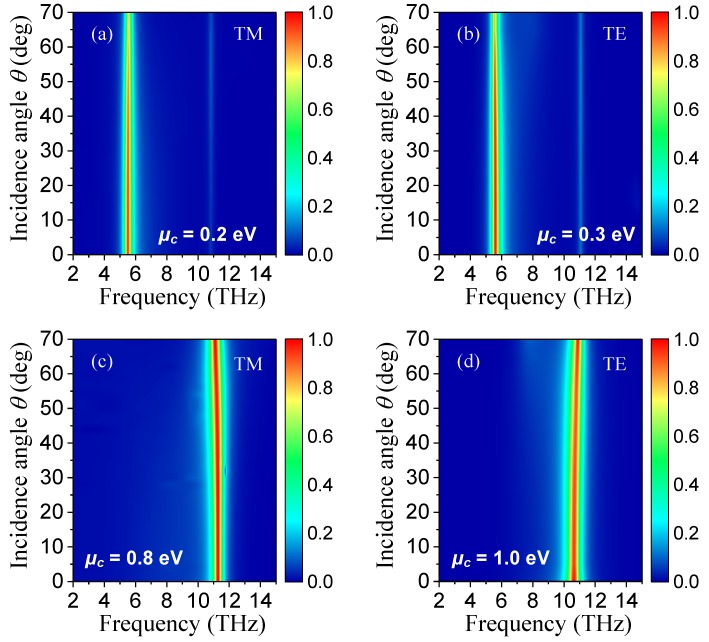
Simulated absorption spectra of the proposed absorber with different incident angles *θ* under the (**a**) TM polarization with *μ*_c_ = 0.2 eV and (**b**) TE polarization with *μ*_c_ = 0.3 eV. (**c**) TM polarization with *μ*_c_ = 0.8 eV and (**d**) TE polarization with *μ*_c_ = 1.0 eV.

Furthermore, we also investigated the angular stability of the proposed absorber with DLDGRAs. Here, for example, we present the simulated absorbance spectra as a function of incidence angle *θ* and operating frequency with *μ*_c_ of 0.2 and 0.8 eV for TM polarization in Figure 7a,c, and with *μ*_c_ of 0.3 and 1.0 eV for TE polarization in Figure 7b,d, respectively. These results show that the absorbance could still maintain above 71.7%, 82.7%, 88.9%, and 96.9% for Figure 7a–d over a wide angle of incidence ranging from 0° to 70°, respectively. Therefore, the proposed absorber with DLDGRAs demonstrated ultra-wide angular stability, which can be mainly attributed to the wide-angle property of localized resonance of SPPs in individual graphene ribbons.

### 3.3. Analytical Prediction of the Reconfigurable Absorbance Peak Frequencies 

**Figure 8:**
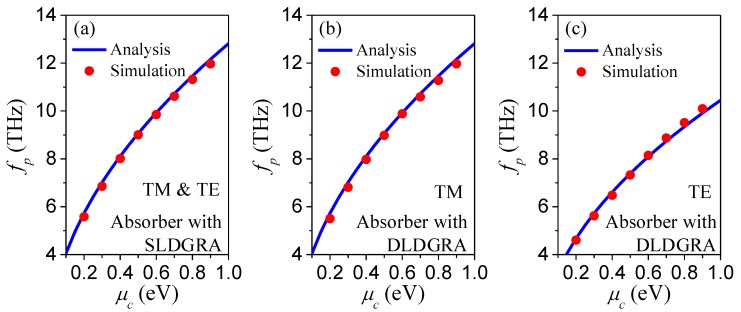
The variation of analytical and simulation absorbance peak frequency with different *μ*_c_ (**a**) for the absorber with a SLDGRA under both TM and TE polarizations; (**b**) for the absorber with DLDGRAs under TM polarization, and (**c**) for the absorber with DLDGRAs under TE polarization.

To predict the reconfigurable frequencies of the proposed absorbers, we further studied the relationship between the absorbance peak frequency *f*_p_ and *μ*_c_. According to the quasi-static analysis in References [39,40], a fitting mode for *f*_p_ and *μ*_c_ is given by $f_{p}=\frac{ke}{\hbar }\sqrt{\frac{\mu_{c}}{W\varepsilon_{e}\varepsilon_{o}}}$, where *k* = 0.1 denotes a dimensionless fitting constant, *W* is the width of graphene ribbon, *ε*_e_ is the effective permittivity of the background materials surrounding the graphene ribbon array, and *ε*_o_ is the permittivity of vacuum, respectively. Here, we first compared the *f*_p_ of the absorber with a SLDGRA obtained from this analytical fitting mode and the simulated results, as shown in Figure 8a. In this case, the *ε*_e_ was expressed as *ε*_e_ = (*ε*_Zro2_ + 1)/2. It was found that with *μ*_c_ varying from 0.2 to 0.9 eV, *f*_p_ increased from 5.58 to 11.97 THz. The analytical fitting results matched well with the simulation results. Then, we verified this analytical fitting mode for the absorber with the DLDGRA case. Because it is polarization-dependent, we compared the fitting and simulation results separately for TM and TE polarizations, as shown in Figure 8b,c. For TM polarization, the terahertz LSPPs predominated on the upper-layer graphene ribbon, as shown in Figure 6a. Therefore, *ε*_e_ was also approximately expressed as *ε*_e_ = (*ε*_Zro2_ + 1)/2. With the same values of *ε*_e_ for the absorber with DLDGRAs under TM polarization and the absorber with SLDGRA, the same analytical predictions of *f*_p_ are shown in both Figure 8a,b. On contrary, the LSPPs mainly concentrated on the lower-layer graphene ribbon for the TE polarization, and *ε*_e_ should be calculated by *ε*_e_ = *ε*_Zro2_ instead. Both analytical prediction and the simulation results clearly showed that *f*_p_ increased from 5.50 (4.60) to 11.97 (10.09) THz for TM (TE) polarization with *μ*_c_ varying from 0.2 to 0.9 eV with good agreement. Therefore, this analytical fitting mode can be used for the efficient prediction of the reconfigurable absorbance peak frequencies of this variety of absorbers.

## 4. Conclusions

We systematically demonstrated a new variety of frequency-reconfigurable absorbers using S/DLDGRAs. The absorbers had excellent properties of narrowband near-unity terahertz absorbance, ultra-wide frequency reconfiguration, and angular stability. For the absorber with a SLDGRA, the over 90% absorbance peak frequency could be flexibly adjusted from 6.85 to 9.85 THz for both polarizations under normal incidence by tuning *μ*_c_ from 0.3 to 0.6 eV. For the absorber with DLDGRAs, the absorbance peak frequencies of each reconfigurable bands could be significantly extended from 5.50 to 11.31 THz and 5.60 to 10.61 THz under TM and TE polarizations, respectively, approaching two octaves. Good angular stability of these absorbers was achieved, with the absorbance peaks of the reconfigurable absorbance bands maintained over 70% in a wide angle of incidence ranging from 0° to 70° for both polarizations. We proposed an analytical fitting model to predict the absorbers’ reconfigurable absorbance peak frequencies. Our results demonstrated that the proposed frequency-reconfigurable absorbers offer significant potential to realize novel reconfigurable optoelectronic devices, which may have great applications in the emerging terahertz regime.
